# Yield of molecular autopsy in sudden cardiac death in athletes: data from a large registry in the UK

**DOI:** 10.1093/europace/euae029

**Published:** 2024-01-30

**Authors:** Gherardo Finocchiaro, Davide Radaelli, David Johnson, Raghav T Bhatia, Joseph Westaby, Stefano D’Errico, Michael Papadakis, Sanjay Sharma, Mary N Sheppard, Elijah R Behr

**Affiliations:** Cardiovascular Clinical Academic Group and Cardiology Research Section, St George’s, University of London, St George’s University Hospitals NHS Foundation Trust, Cranmer Terrace, London SW17 0RE, UK; Cardiovascular Clinical Academic Group and Cardiology Research Section, St George’s, University of London, St George’s University Hospitals NHS Foundation Trust, Cranmer Terrace, London SW17 0RE, UK; Department of Medicine, Surgery and Health, University of Trieste, Trieste, Italy; Cardiovascular Clinical Academic Group and Cardiology Research Section, St George’s, University of London, St George’s University Hospitals NHS Foundation Trust, Cranmer Terrace, London SW17 0RE, UK; Cardiovascular Clinical Academic Group and Cardiology Research Section, St George’s, University of London, St George’s University Hospitals NHS Foundation Trust, Cranmer Terrace, London SW17 0RE, UK; Cardiovascular Clinical Academic Group and Cardiology Research Section, St George’s, University of London, St George’s University Hospitals NHS Foundation Trust, Cranmer Terrace, London SW17 0RE, UK; Department of Medicine, Surgery and Health, University of Trieste, Trieste, Italy; Cardiovascular Clinical Academic Group and Cardiology Research Section, St George’s, University of London, St George’s University Hospitals NHS Foundation Trust, Cranmer Terrace, London SW17 0RE, UK; Cardiovascular Clinical Academic Group and Cardiology Research Section, St George’s, University of London, St George’s University Hospitals NHS Foundation Trust, Cranmer Terrace, London SW17 0RE, UK; Cardiovascular Clinical Academic Group and Cardiology Research Section, St George’s, University of London, St George’s University Hospitals NHS Foundation Trust, Cranmer Terrace, London SW17 0RE, UK; Cardiovascular Clinical Academic Group and Cardiology Research Section, St George’s, University of London, St George’s University Hospitals NHS Foundation Trust, Cranmer Terrace, London SW17 0RE, UK

**Keywords:** Molecular autopsy, Sudden cardiac death

## Abstract

**Aims:**

Sudden cardiac death (SCD) may occur in apparently healthy individuals, including athletes. The aim was to investigate the diagnostic role of post-mortem genetic testing, molecular autopsy (MA), in elucidating the cause of SCD in athletes.

**Methods and results:**

We reviewed a database of 6860 consecutive cases of SCD referred to our specialist cardiac pathology centre. All cases underwent detailed cardiac autopsy, and 748 were deemed to be athletes. Of these, 42 (6%) were investigated with MA (28 using a targeted sequencing, 14 exome sequencing). Variants were classified as pathogenic, likely pathogenic, or variant of unknown significance using international guidelines. Clinical information was obtained from referring coroners who completed a detailed health questionnaire. Out of the 42 decedents (average age 35 years old, 98% males) who were investigated with MA, the autopsy was in keeping with a structurally normal heart [sudden arrhythmic death syndrome (SADS)] in *n* = 33 (78%) cases, followed by arrhythmogenic cardiomyopathy (ACM) in eight (19%) individuals and idiopathic left ventricular fibrosis in one (2%). Death occurred during exercise and at rest in 26 (62%) and 16 (38%) individuals, respectively. Variants that were adjudicated clinically actionable were present in seven cases (17%). There was concordance between the genetic and phenotypic findings in two cases of ACM (in FLNC and TMEM43 genes). None of the variants identified in SADS cases were previously linked to channelopathies. Clinically actionable variants in cardiomyopathy-associated genes were found in five cases of SADS.

**Conclusion:**

The yield of MA in athletes who died suddenly is 17%. In SADS cases, clinically actionable variants were found in cardiomyopathy-associated genes and not in channelopathy-associated genes. Arrhythmogenic cardiomyopathy is a common cause of SCD in athletes, and one in four decedents with this condition had a clinically actionable variant in FLNC and TMEM43 genes.

Sudden cardiac death (SCD) may occur in apparently healthy individuals including athletes.

The yield of post-mortem genetic testing [molecular autopsy (MA)] in athletes who died suddenly is unknown.^1^ We aimed to assess the yield of pathogenic (P) and likely pathogenic (LP) variants by MA in a cohort of athletes who died suddenly and underwent post-mortem examination by an expert cardiac pathologists.

We reviewed a database of 6860 consecutive cases of SCD referred to our specialist cardiac pathology centre at St George’s, University of London, between 1994 and 2020. Sudden cardiac death was defined as death from a cardiovascular cause within 1 h of onset of symptoms if witnessed or within 12 h of onset if unwitnessed. Clinical information was obtained from referring coroners who were asked to complete a detailed health questionnaire. We arbitrarily defined athletes as individuals that engaged in at least 5 hours of organized exercise activity per week. All cases underwent detailed post-mortem evaluation of the heart, including histological analysis, by expert cardiac pathologists (M.N.S., J.W.). A minimum of 10 blocks of tissue were taken for histological analysis, and cardiomyopathy was defined as reported previously.^1,2^ Death during exercise was defined as occurring while the individual was engaging in exercise, as opposed to death during daily activities or rest. Molecular autopsy was performed with a targeted panel and whole exome sequencing focusing on a broad panel of genes implicated in channelopathy and cardiomyopathy as previously described.^3^ Variants were classified manually as P, LP, or a variant of unknown significance (VUS) using the American College of Medical Genetics and Genomics (ACMG) consensus statement guidelines.^4^ Ethical and research governance approval have been granted for this study (10/H0724/38). Results are expressed as mean ± standard deviation (SD) for continuous variables or as number of cases and percentage for categorical variables.

Out of the total cohort, 748 individuals were athletes. A minority of consecutive athletes with DNA available (*n* = 42, 6%) were investigated with MA: 28 individuals with targeted panel sequencing and 14 with whole exome sequencing. The average age was 35 ± 11 years old and 98% (*n* = 41) were male. A structurally normal heart at the post-mortem examination with negative toxicology [sudden arrhythmic death syndrome (SADS)] was found in 33 (78%) decedents. Arrhythmogenic cardiomyopathy (ACM) was observed in eight (19%) cases and idiopathic left ventricular fibrosis in one (2%). Death occurred during exercise in 26 (62%) cases and at rest in 16 (38%), including five (12%) cases where death occurred during sleep. Molecular autopsy showed P/LP variants in nine (21%) individuals. Variants that were adjudicated as the likely cause of death were present in seven cases (17%). Two cases had a P/LP variant in *HFE* in heterozygous state unlikely to cause haemochromatosis. There was concordance between the genetic and phenotypic findings in two cases of ACM (*Table 1*). No P/LP variants linked to channelopathies were identified in the SADS cases (*Table 1*).

**Table 1:** Genetic test results in athletes who died suddenly

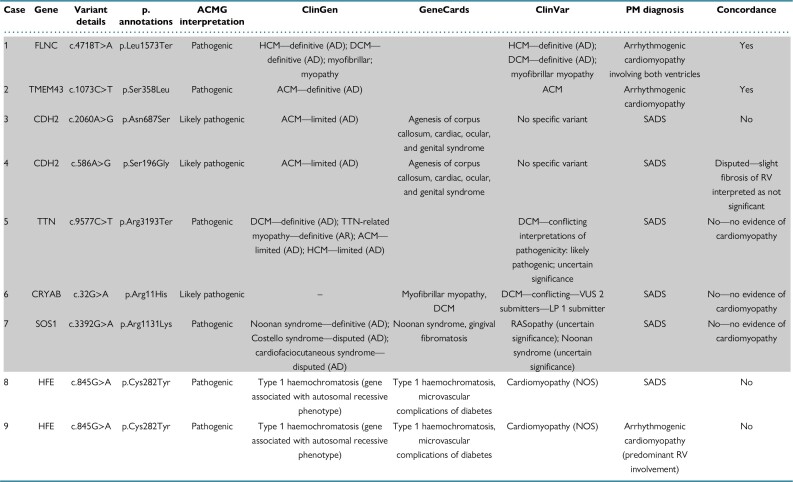

All variants were heterozygous. The first seven cases (in shaded) are deemed to harbour an actionable variant. The ‘Concordance’ column indicates whether there is concordance between the genetic data and the morphological findings.

ACM, arrhythmogenic cardiomyopathy; ACMG, American College of Medical Genetics and Genomics; AD, autosomal dominant; AR, autosomal recessive; ClinGen, Clinical Genome Resource (https://clinicalgenome.org/); ClinVar, NCBI database (https://www.ncbi.nlm.nih.gov/clinvar/); DCM, dilated cardiomyopathy; HCM, hypertrophic cardiomyopathy; LP, likely pathogenic; NOS, not otherwise specified; PM, post-mortem; RV, right ventricle; SADS, sudden arrhythmic death syndrome; VUS, variant of uncertain unknown significance.

Athletes often appear to epitomize health, but SCD may occur in apparently healthy individuals.^1,5^ Post-mortem examination is a crucial diagnostic step in establishing the cause of SCD and in guiding the clinical evaluation of surviving relatives.^6,7,8,9^ The interpretation of the post-mortem results, however, can be challenging especially when the heart is structurally normal or when abnormalities of uncertain significance are found.^10^ Molecular autopsy has the potential to establish the cause of death.^11,12^ Indeed, a study from our group on a large cohort of SADS decedents investigated with MA found clinically actionable P or LP variants in 13% of the cases, mostly associated with channelopathy.^3^ Our study comprised 42 athletes where the post-mortem examination performed by expert cardiac pathologists was mostly in keeping with a structurally normal heart and ACM. Roughly one-fifth of athletes had a P or LP variant. Clinically actionable variants were found in 17% of cases (*Figure 1*). Interestingly, none of the athletes with SADS were found to have P/LP variants associated with channelopathies. In all cases, P/LP variants were identified in cardiomyopathy-associated genes. This suggests that cardiomyopathy, even when ‘concealed’ and not detected at expert cardiac autopsy, may predispose to SCD in young male athletes. This is in line with a recent study on 91 autopsy-inconclusive SCD cases where cardiomyopathy-associated genes harboured 70% of clinically actionable variants.^13^ Genetic findings correlated with the phenotype in only two cases, both with ACM; *FLNC* and *TMEM43* were the genes involved, which emphasizes the arrhythmic risk of these specific disorders.^14,15^ Although our cohort is large, not all cases of SCD in the UK are referred to our centre and this introduces a bias. Further, only 6% of athletes who died suddenly and were referred to our centre were investigated with MA. This implies a potential selection bias that should be taken into account when interpreting the results.

**Figure 1:**
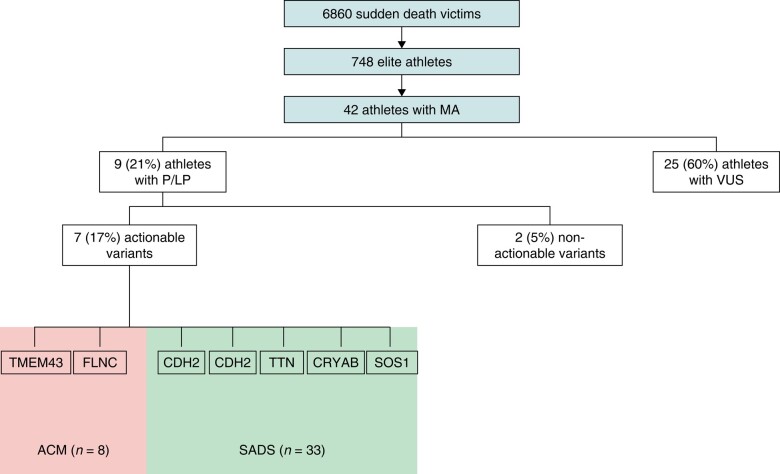
Molecular autopsy in sudden cardiac death in athletes. Genes involved are described. Seven actionable variants have been found in seven athletes. ACM, arrhythmogenic cardiomyopathy; LP, likely pathogenic variant; MA, molecular autopsy; P, pathogenic; SADS, sudden arrhythmic death syndrome; VUS, variant of unknown significance.

In conclusion, in a small cohort of athletes who died suddenly and who were investigated with post-mortem genetic test, the yield of MA was 17%. In SADS cases, P/LP variants were found in cardiomyopathy-associated genes and not in channelopathy-associated genes. Genetic panels should include assessment of genes implicated in cardiomyopathy even when a clear phenotype is not identified through post-mortem examination. One in four decedents with ACM was identified with P/LP variants.

## Data Availability

Data supporting this study are available from CRY Centre for Cardiac Pathology in St George’s University, London. Access to the data is subject to approval and a data sharing agreement due to ethical reason.
